# Emergence of Non-Fourier Hierarchies

**DOI:** 10.3390/e20110832

**Published:** 2018-10-30

**Authors:** Tamás Fülöp, Róbert Kovács, Ádám Lovas, Ágnes Rieth, Tamás Fodor, Mátyás Szücs, Péter Ván, Gyula Gróf

**Affiliations:** 1 Department of Energy Engineering, Faculty of Mechanical Engineering, BME, 1521 Budapest, Hungary; 2 Montavid Thermodynamic Research Group, 1112 Budapest, Hungary; 3 Department of Theoretical Physics, Institute for Particle and Nuclear Physics, Wigner Research Centre for Physics, 1525 Budapest, Hungary

**Keywords:** non-Fourier heat conduction, thermal expansion, heat pulse experiments, pseudo-temperature, Guyer-Krumhansl equation

## Abstract

The non-Fourier heat conduction phenomenon on room temperature is analyzed from various aspects. The first one shows its experimental side, in what form it occurs, and how we treated it. It is demonstrated that the Guyer-Krumhansl equation can be the next appropriate extension of Fourier’s law for room-temperature phenomena in modeling of heterogeneous materials. The second approach provides an interpretation of generalized heat conduction equations using a simple thermo-mechanical background. Here, Fourier heat conduction is coupled to elasticity via thermal expansion, resulting in a particular generalized heat equation for the temperature field. Both aforementioned approaches show the size dependency of non-Fourier heat conduction. Finally, a third approach is presented, called pseudo-temperature modeling. It is shown that non-Fourier temperature history can be produced by mixing different solutions of Fourier’s law. That kind of explanation indicates the interpretation of underlying heat conduction mechanics behind non-Fourier phenomena.

## 1. Introduction

The Fourier’s law [1]

(1) $$\begin{array}{c} q=-k\overset{\to }{\nabla }T \end{array}$$

is one of the most applicable, well-known elementary physical laws in engineering practice. Here, q is the heat flux vector, *T* is absolute temperature, *k* is thermal conductivity. However, as all the constitutive equations, it also has limits of validation. Phenomena that do not fit into these limits, called non-Fourier heat conduction, appear in many different forms. Some of them occur at low temperature such as the so-called second sound and ballistic (thermal expansion induced) propagation [2,3,4,5,6,7]. These phenomena have been experimentally measured several times [8,9,10,11] and many generalized heat equations exist to simulate them [12,13,14,15,16,17,18,19,20]. The success in low-temperature experiments resulted in the extension of this research field to find the deviation at room temperature as well. One of the most celebrated result is related to Mitra et al. [21,22] where the measured temperature history was very similar to a wave-like propagation. However, these results have not been reproduced by anyone and undoubtedly demanded for further investigation.

In most of the room-temperature measurements, the existence of Maxwell-Cattaneo-Vernotte (MCV) type behavior attempted to be proved [23,24]. It is this MCV equation that is used to model the aforementioned second sound, the dissipative wave propagation form of heat [3,25,26]. The validity of MCV equation for room-temperature behavior has not yet been justified, despite of the numerous experiments. It is important to note that many other extensions of Fourier equation exist beyond the MCV one, such as the Guyer-Krumhansl (GK) equation [27,28,29,30,31,32], the dual-phase-lag model [33], and their modifications, too [7,34,35]. Some of these possess stronger physical background, some others not [36,37,38]. Here we would like to emphasize that we restrict ourselves to the GK equation that shows the simplest hierarchical arrangement of Fourier’s law and applicable for room-temperature problems.

The simplest extension of MCV equation is the GK model, which reads:

(2) $$\begin{array}{c} \tau \dot{q}+q+k\overset{\to }{\nabla }T-\kappa^{2}▵q=0, \end{array}$$

where the coefficient τ is called relaxation time and $\kappa^{2}$ is regarded as a dissipation parameter and the dot denotes the time derivative. This GK-type constitutive equation contains the MCV-type by considering $\kappa^{2}=0$ and the Fourier equation taking $\tau =\kappa^{2}=0$. This feature of GK equation allows to model both wave-like temperature history and over-diffusive one. This is more apparent when one applies the balance equation of internal energy to eliminate q:

(3) $$\begin{array}{c} \rho c\dot{T}+\overset{\to }{\nabla }\cdot q=0, \end{array}$$

with mass density ρ, specific heat *c* and volumetric source neglected, one obtains

(4) $$\begin{array}{c} \tau \ddot{T}+\dot{T}=a▵T+\kappa^{2}▵\dot{T}, \end{array}$$

with thermal diffusivity a=k/(ρc). One can realize that Equation (4) contains the Fourier heat equation

(5) $$\begin{array}{c} \dot{T}=a▵T \end{array}$$

as well as its time derivative, with different coefficients. It becomes more visible after rearranging Equation (4):

(6) $$\begin{array}{c} \tau {\left(\dot{T}-\frac{\kappa^{2}}{\tau }▵T\right)}^{.}+\dot{T}-a▵T=0. \end{array}$$

when the so-called [39,40] Fourier resonance condition $\kappa^{2}/\tau =a$ holds, the solutions of the Fourier Equation (5) are covered by the solutions of (4). Meanwhile, when $\kappa^{2}<a\tau$ the wave-like behavior is recovered, and this domain is known as under-damped region. In the opposite case ($\kappa^{2}>a\tau$), there is no visible wave propagation and it is called over-diffusive (or over-damped) region. We measured the corresponding over-diffusive effect several times in various materials such as metal foams, rocks and in a capacitor, too [39,40]. Furthermore, a similar temperature history was observed in a biological material [38]. It is also important to note that originally the GK equation is derived from Boltzmann equation applying phonon hydrodynamics in the background. Here, we would like to emphasize that in non-equilibrium thermodynamics it can also be derived without assuming any phonon interaction in the material [6,7] keeping the GK equation applicable for room-temperature heat conduction.

In this paper, further aspects of over-diffusive propagation are discussed. In the following sections the size dependence of the observed over-damped phenomenon is discussed both experimentally and theoretically. Moreover, the approach of pseudo-temperature is presented to provide one concrete possible interpretation for non-Fourier heat conduction.

## 2. Size Dependence

**Figure 1:**
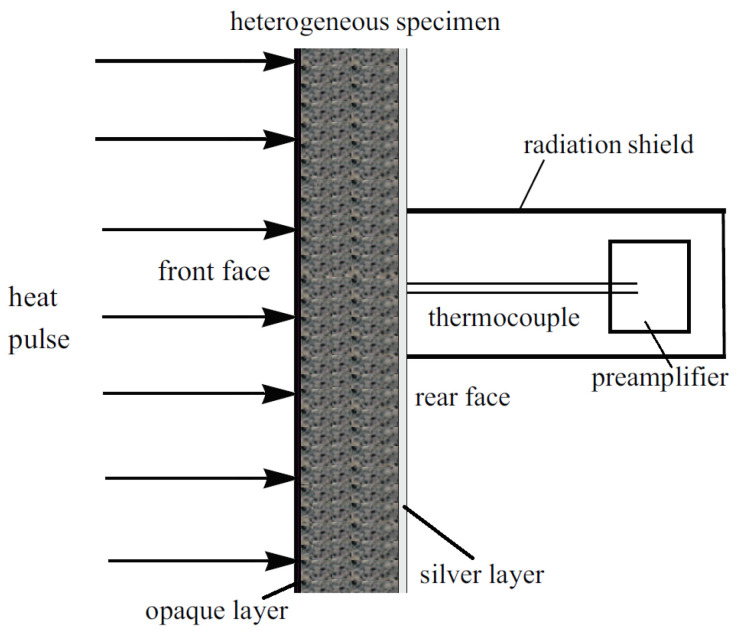
Setup of our heat pulse experiment [40].

Our measurements reported here are performed on basalt rock samples with three different thicknesses, 1.86, 2.75 and 3.84 mm, respectively. We have applied the same apparatus of heat pulse experiment as described in [39,40], schematically depicted in Figure 1 below.

**Figure 2:**
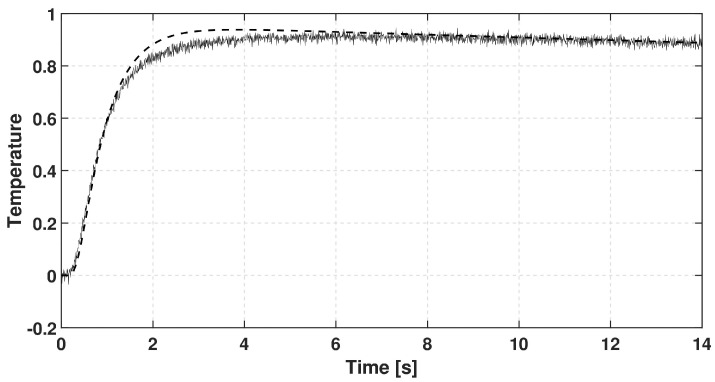
Data recorded for basalt rock sample with thickness of 1.86 mm. The dashed line shows the prediction of Fourier’s law.

**Figure 3:**
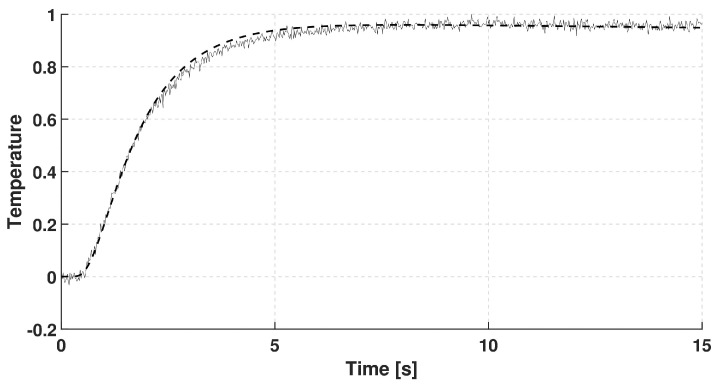
Data recorded for basalt rock sample with thickness of 2.75 mm. The dashed line shows the prediction of Fourier’s law.

**Figure 4:**
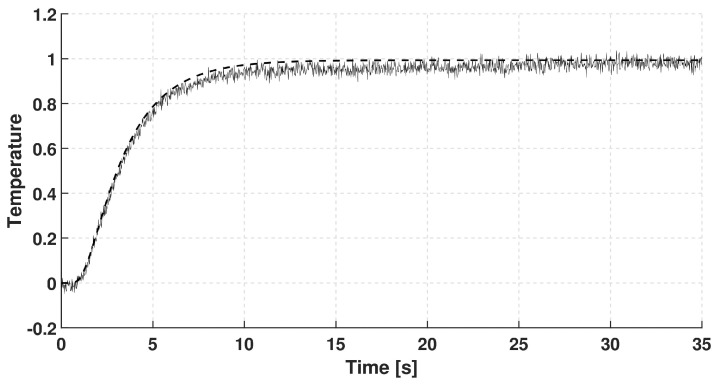
Data recorded for basalt rock sample with thickness of 3.84 mm. The dashed line shows the prediction of Fourier’s law.

In each case, the rear-side temperature history was measured and numerically evaluated solving the GK equation with constant coefficients, i.e., they do not depend on the temperature due to its small change. It is also assumed that the GK equation characterizes the whole sample. We choose the GK equation as the simplest thermodynamically consistent one that can predict signal shapes observed in room-temperature measurements. (The heat pulse setup—a widely used one for transient heat conduction measurements—is not capable of obtaining space dependence of temperature along the sample but even such measurement data would be insufficient to determine an underlying partial differential equation - any experimental data can only refute or support an equation (at some confidence level).) The GK coefficients used below are best fits. The recorded dimensionless temperature signals are plotted in Figure 2, Figure 3 and Figure 4. In these figures, the dashed line shows the solution of Fourier equation using thermal diffusivity corresponding to the initial part of temperature rising on the rear side. The measured signal deviates from the Fourier-predicted one even when considering non-adiabatic (cooling) boundary condition. That deviation weakens with increasing sample thickness; for the thickest one it is hardly visible, and the prediction of Fourier’s law is almost acceptable.

**Figure 5:**
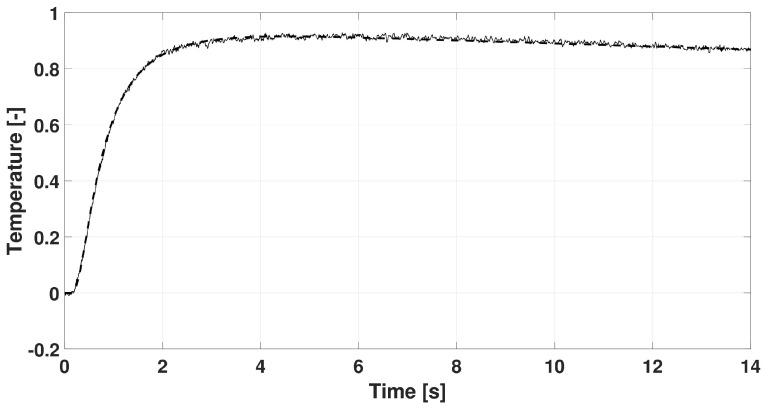
Data recorded using the basalt with thickness of 1.86 mm. The dashed line shows the prediction of GK equation.

The evaluation of the thinnest sample using the GK equation is shown in Figure 5. The fitted coefficients are summarized in Table 1. It is important to mention that MCV equation using the presented parameters would show a wave-like propagation that is not observed in the experiments.

Deviation from the Fourier prediction is weak but is clearly present, and has size dependent attributes. Concerning the ratio of parameters, i.e., investigating how considerably the Fourier resonance condition $a\tau /\kappa^{2}=1$ is violated, the outcome can be seen in Table 2. As analysis of the results, it is remarkable to note the deviation of the GK fitted thermal diffusivity from the Fourier fitted one, and that this deviation is size dependent. For the thickest sample, which can be well described by Fourier’s law, the fitted thermal diffusivity values are practically equal, and the ratio of parameters is very close to the Fourier resonance value 1.

The next section is devoted to a possible explanation for the emergence of a generalized heat equation with higher time and space derivatives. All coefficients of the higher time and space derivative terms are related to well-known material parameters. The result also features size dependent non-Fourier deviation.

## 3. Seeming Non-Fourier Heat Conduction Induced by Elasticity Coupled via Thermal Expansion

While, in general, one does not have a direct physical interpretation of the phenomenon that leads to, at the phenomenological level, non-Fourier heat conduction here follows a case where we do know this background phenomenon. Namely, in case of heat conduction in solids, a plausible possibility is provided by an interplay between elasticity and thermal expansion. Namely, without thermal expansion, elasticity—a tensorial behavior—is not coupled to Fourier heat conduction—a vectorial one—in isotropic materials. However, with nonzero thermal expansion, strains and displacements must be in accord both with what elastic mechanics dictates and with what position dependent temperature imposes. The coupled set of equations of Fourier heat conduction, of elastic mechanics and of kinematic relationships, after eliminating the kinematic and mechanical quantities, leads to an equation for temperature only that contains higher derivative corrections to Fourier’s equation. It is important to check how remarkable these corrections are. In the following section we present this derivation and investigation.

### The Basic Equations

In all respects involved, we choose the simplest assumptions: the small-strain regime, a Hooke-elastic homogeneous and isotropic solid material, with constant thermal expansion coefficient, essentially being at rest with respect to an inertial reference frame. Kinematic, mechanical and thermodynamical quantities and their relationships are considered along the approach detailed in [41,42,43].

The Hooke-elastic homogeneous and isotropic material model states, at any position **r**, the constitutive relationship

(7) $$\begin{array}{c} \sigma^{d}=E^{d}D^{d},\;\sigma^{s}=E^{s}D^{s},\;\;E^{d}=2G,\;E^{s}=3K, \end{array}$$

(8) $$\begin{array}{c} \sigma =E^{d}D^{d}+E^{s}D^{s}=E^{d}D+\left(E^{s}-E^{d}\right)D^{s} \end{array}$$

between stress tensor σ and elastic deformedness tensor **D** (which, in many cases, coincides with the strain tensor), where ${}^{d}$ and ${}^{s}$ denote the deviatoric (traceless) and spherical (proportional to the unit tensor **1**) parts, i.e.,

(9) $$\begin{array}{c} D^{s}=\frac{1}{3}\left(\mathrm{tr}\;D\right)1,\;D^{d}=D-D^{s};\;\mathrm{hence},\;e.g.,\;1^{s}=1,\;1^{d}=0. \end{array}$$

Stress induces a time derivative in the velocity field v of the solid medium, according to the equation

(10) $$\begin{array}{c} \varrho \dot{v}=\sigma \;\cdot \;\overset{\leftarrow }{\nabla } \end{array}$$

with mass density ϱ being constant in the in the small-strain regime; hereafter $\overset{\leftarrow }{\nabla }$ and $\overset{\to }{\nabla }$ denote derivative of the function standing to the left and to the right, respectively, to display the tensorial order (tensorial index order) properly for vector/tensor valued functions. For the velocity gradient L and its symmetric part, one has

(11) $$\begin{array}{c} L=v⊗\overset{\leftarrow }{\nabla },\;\mathrm{tr}\;L^{\mathrm{sym}}=\mathrm{tr}\;L=v\;\cdot \;\overset{\leftarrow }{\nabla },\;{\left(L^{\mathrm{sym}}\right)}^{s}=\frac{1}{3}\left(\mathrm{tr}\;L^{\mathrm{sym}}\right)1=\frac{1}{3}\left(v\;\cdot \;\overset{\leftarrow }{\nabla }\right)1, \end{array}$$

(12) $$\begin{array}{cc} \left(L^{\mathrm{sym}}\;\cdot \;\overset{\leftarrow }{\nabla }\right)\;\cdot \;\overset{\leftarrow }{\nabla } & =\frac{1}{2}\partial_{i}\partial_{j}\left(\partial_{i}v_{j}+\partial_{j}v_{i}\right)=\frac{1}{2}\left[▵\left(\overset{\to }{\nabla }\;\cdot \;v\right)+▵\left(\overset{\to }{\nabla }\;\cdot \;v\right)\right]=▵\left(v\;\cdot \;\overset{\leftarrow }{\nabla }\right), \end{array}$$

(13) $$\begin{array}{cc} \left(L\;\cdot \;\overset{\leftarrow }{\nabla }\right)\;\cdot \;\overset{\leftarrow }{\nabla } & =▵\left(v\;\cdot \;\overset{\leftarrow }{\nabla }\right), \end{array}$$

where the Einstein summation convention for indices has also been applied. Again, using this convention, and the Kronecker delta notation, to any scalar field *f*,

(14) $$\begin{array}{c} \partial_{j}\left(f\delta_{ij}\right)=\delta_{ij}\partial_{j}f=\partial_{i}f,\;\left(f1\right)\;\cdot \;\overset{\leftarrow }{\nabla }=\overset{\to }{\nabla }f \end{array}$$

follow, which are also to be used below.

The small-deformedness relationship among the kinematic quantities, with linear thermal expansion coefficient α considered constant, and absolute temperature *T*, is

(15) $$\begin{array}{c} L^{\mathrm{sym}}=\dot{D}+\alpha \dot{T}1\;. \end{array}$$

For specific internal energy *e*,

(16) $$\begin{array}{c} e=cT+\frac{E^{s}\alpha }{\varrho }T\mathrm{tr}\;D^{s}+e_{\mathrm{el}}\;,\;e_{\mathrm{el}}=\frac{E^{d}}{2\varrho }\mathrm{tr}\;\left[{\left(D^{d}\right)}^{2}\right]+\frac{E^{s}}{2\varrho }\mathrm{tr}\;\left[{\left(D^{s}\right)}^{2}\right]\;, \end{array}$$

its balance,

(17) $$\begin{array}{c} \varrho \dot{e}=\mathrm{tr}\;\left(\sigma L\right)-q\;\cdot \;\overset{\leftarrow }{\nabla }\;, \end{array}$$

after subtracting the contribution $\varrho {\dot{e}}_{\mathrm{el}}$ coming from specific elastic energy $e_{\mathrm{el}}$ and the corresponding elastic part $\mathrm{tr}\;\left(\sigma \dot{D}\right)$ of the mechanical power $\mathrm{tr}\;\left(\sigma L\right)$, is

(18) $$\begin{array}{c} \varrho {\left(e-e_{\mathrm{el}}\right)}^{\;\cdot }=\varrho c\dot{T}+E^{s}\alpha T_{0}\mathrm{tr}\;{\dot{D}}^{s}=-q\;\cdot \;\overset{\leftarrow }{\nabla }\;,\;\mathrm{with}\;q=-k\overset{\to }{\nabla }T\;, \end{array}$$

where *c* is specific heat corresponding to constant zero stress (or pressure), temperature has been approximated in one term of (18) by an initial homogeneous absolute temperature value $T_{0}$ to stay in accord with the linear (small-strain) approximation, and heat flux **q** follows the Fourier heat conduction constitutive relationship with thermal conductivity *k* also treated as a constant.

#### The Derivation

The strategy is to eliminate σ in favor of (with the aid of) **D**, then **D** is eliminated in favor of $L^{\mathrm{sym}}$, after which we can realize that both from the mechanical direction and from the thermal one we obtain relationship between $v\;\cdot \;\overset{\leftarrow }{\nabla }$ and *T*, which, eliminating $v\;\cdot \;\overset{\leftarrow }{\nabla }$, yields an equation for *T* only.

Starting with the thermal side,

(19) $$\begin{array}{cc} \varrho c\dot{T}+E^{s}\alpha T_{0}\mathrm{tr}\;{\left(L^{\mathrm{sym}}-\alpha \dot{T}1\right)}^{s} & =\varrho c\dot{T}+E^{s}\alpha T_{0}\left(v\;\cdot \;\overset{\leftarrow }{\nabla }\right)-E^{s}\alpha^{2}T_{0}\dot{T}\cdot 3=\\ & =(\underset{\gamma_{1}^{}}{\underset{︸}{\varrho c-3E^{s}\alpha^{2}T_{0}}})\dot{T}+E^{s}\alpha T_{0}\left(v\;\cdot \;\overset{\leftarrow }{\nabla }\right)\;,\\ & =-q\;\cdot \;\overset{\leftarrow }{\nabla }=-\left(-k\overset{\to }{\nabla }T\right)\;\cdot \;\overset{\leftarrow }{\nabla }=k▵T\;⟹ \end{array}$$

(20) $$\begin{array}{cc} E^{s}\alpha T_{0}\left(v\;\cdot \;\overset{\leftarrow }{\nabla }\right) & =k▵T-\gamma_{1}^{}\dot{T}\;. \end{array}$$

Meanwhile, from the mechanical direction, aiming at being in tune with (20):

$$\begin{array}{cc} E^{s}\alpha T_{0}\left(\ddot{v}\cdot \overset{\leftarrow }{\nabla }\right) & =E^{s}\alpha T_{0}\frac{1}{\varrho }\left(\dot{\sigma }\cdot \overset{\leftarrow }{\nabla }\right)\cdot \overset{\leftarrow }{\nabla }=\\ & =\frac{E^{s}\alpha T_{0}}{\varrho }\left\{\left[E^{d}\dot{D}+\left(E^{s}-E^{d}\right){\dot{D}}^{s}\right]\cdot \overset{\leftarrow }{\nabla }\right\}\cdot \overset{\leftarrow }{\nabla }=\\ & =\frac{E^{s}\alpha T_{0}}{\varrho }\{[E^{d}\left(L^{\mathrm{sym}}-\alpha \dot{T}1\right)+\\ & \;+\left(E^{s}-E^{d}\right){\left(L^{\mathrm{sym}}-\alpha \dot{T}1\right)}^{s}]\cdot \overset{\leftarrow }{\nabla }\}\cdot \overset{\leftarrow }{\nabla }=\\ & =\frac{E^{s}\alpha T_{0}}{\varrho }\{[E^{d}L^{\mathrm{sym}}-E^{d}\alpha \dot{T}1+\left(E^{s}-E^{d}\right)\frac{1}{3}\left(v\;\cdot \;\overset{\leftarrow }{\nabla }\right)1-\\ & \;-\left(E^{s}-E^{d}\right)\alpha \dot{T}1]\cdot \overset{\leftarrow }{\nabla }\}\cdot \overset{\leftarrow }{\nabla }=\\ & =\frac{E^{s}\alpha T_{0}}{\varrho }\left[E^{d}▵\left(v\;\cdot \;\overset{\leftarrow }{\nabla }\right)+\frac{E^{s}-E^{d}}{3}▵\left(v\;\cdot \;\overset{\leftarrow }{\nabla }\right)-E^{s}\alpha ▵\dot{T}\right]=\\ & =\frac{E^{s}\alpha T_{0}}{\varrho }\left[\frac{E^{s}+2E^{d}}{3}▵\left(v\;\cdot \;\overset{\leftarrow }{\nabla }\right)-E^{s}\alpha ▵\dot{T}\right]=\\ & =\frac{E^{s}+2E^{d}}{3\varrho }▵\left[E^{s}\alpha T_{0}\left(v\;\cdot \;\overset{\leftarrow }{\nabla }\right)\right]-\frac{{\left(E^{s}\alpha \right)}^{2}T_{0}}{\varrho }▵\dot{T}=\\ & =\underset{c_{\|}^{2}}{\underset{︸}{\frac{E^{s}+2E^{d}}{3\varrho }}}▵\left(k▵T-\gamma_{1}^{}\dot{T}\right)-\frac{{\left(E^{s}\alpha \right)}^{2}T_{0}}{\varrho }▵\dot{T}\;;\;\mathrm{in}\;\mathrm{parallel}, \end{array}$$

(21) $$\begin{array}{cc} & ={\left(k▵T-\gamma_{1}^{}\dot{T}\right)}^{\;\cdot \cdot }=k▵\ddot{T}-\gamma_{1}^{}\dddot{T}\;[\mathrm{cf}.\;(20)] \end{array}$$

(where $c_{\|}$ is the longitudinal elastic wave propagation velocity); hence, summarizing the final result in two equivalent forms,

(22) $$\begin{array}{cc} {\left(\gamma_{1}^{}\dot{T}-k▵T\right)}^{\;\cdot \cdot } & =c_{\|}^{2}▵\left(\underline{\gamma_{1}^{}}\dot{T}-k▵T\right)+\frac{{\left(E^{s}\alpha \right)}^{2}T_{0}}{\varrho }▵\dot{T}\;, \end{array}$$

(23) $$\begin{array}{cc} \gamma_{1}^{}{\left(\ddot{T}-\underline{c_{\|}^{2}}▵T\right)}^{\;\cdot } & =k▵\gamma_{1}^{}\left(\ddot{T}-c_{\|}^{2}▵T\right)+\frac{{\left(E^{s}\alpha \right)}^{2}T_{0}}{\varrho }▵\dot{T}\;. \end{array}$$

The first form here tells us that we have here the wave equation of a heat conduction equation, the last term on the r.h.s. somewhat detuning the heat conduction equation of the r.h.s. with respect to the one on the l.h.s. (the underlined coefficient is the one becoming modified when its term is melted together with the last term). In the meantime, the second form shows the heat conduction equation of a wave equation, the last term on the r.h.s. detuning the underlined coefficient.

Both forms show that coupling, after elimination, leads to a hierarchy of equations, with an amount of detuning that is induced by the coupling—for similar further examples, see [44].

We close this section by rewriting the final result in a form that enables to estimate the contribution of thermal expansion coupled elasticity to heat conduction:

(24) $$\begin{array}{cc} \frac{1}{c_{\|}^{2}}{\left(\gamma_{1}^{}\dot{T}-k▵T\right)}^{\;\cdot \cdot } & =▵\left[\left(\gamma_{1}^{}+\frac{{\left(E^{s}\alpha \right)}^{2}T_{0}}{\varrho c_{\|}^{2}}\right)\dot{T}-k▵T\right]\;, \end{array}$$

i.e.,

(25) $$\begin{array}{cc} \frac{1}{c_{\|}^{2}}{\left(\gamma_{1}^{}\dot{T}-k▵T\right)}^{\;\cdot \cdot } & =▵[\underset{\gamma_{2}^{}}{\left(\underset{︸}{\varrho c-\frac{6E^{d}E^{s}\alpha^{2}T_{0}}{E^{s}+2E^{d}}}\right)}\dot{T}-k▵T]\;. \end{array}$$

One message here is that, thermal expansion coupled elasticity modifies the thermal diffusivity a=k/(ϱc) to an effective one $a_{2}=k/\gamma_{2}^{}=\left(\varrho c/\gamma_{2}^{}\right)\cdot a$ (see the heat conduction on the r.h.s.). For metals, this means a few percent shift (1% for steel and copper, and 6% for aluminum) at room temperature.

The other is that, for a length scale (e.g., characteristic sample size) *ℓ* and the corresponding Fourier time scale $\ell^{2}/a$, the r.h.s. is, to a (very) rough estimate, $1/\ell^{2}$ times a heat conduction equation while the l.h.s. is (similarly roughly)

(26) $$\begin{array}{c} \frac{1}{{\left(\ell^{2}/a\right)}^{2}}\cdot \frac{1}{c_{\|}^{2}} \end{array}$$

times the (nearly) same heat conduction equation (a one with $a_{1}=k/\gamma_{1}^{}$). In other words, the l.h.s. provides a contribution to the r.h.s. via a dimensionless factor

(27) $$\begin{array}{c} \frac{\ell^{2}}{{\left(\ell^{2}/a\right)}^{2}}\cdot \frac{1}{c_{\|}^{2}}=\frac{a^{2}}{\ell^{2}c_{\|}^{2}}. \end{array}$$

This dimensionless factor is about $10^{-10}$ to $10^{-13}$ for metals, $10^{-14}$ for rocks and $10^{-15}$ for plastics with ℓ=3mm, a typical size for flash experiments. Therefore, the effect of the l.h.s. appears to be negligible with respect to the r.h.s.

It is important to point out that the first phenomenon—the emergence of effective thermal diffusivity—would remain unnoticed in the analogous one space dimensional calculation:

(28) $$\begin{array}{cc} \sigma =ED,\;\varrho \dot{v} & =\sigma^{\prime },\;L=v^{\prime }=\dot{D}+\alpha \dot{T}, \end{array}$$

(29) $$\begin{array}{cc} q=-kT^{\prime },\;e & =cT+\frac{E\alpha }{\varrho }TD+\frac{E}{2\varrho }D^{2}\;⟹ \end{array}$$

(30) $$\begin{array}{cc} E{\left[\left(\varrho c-E\alpha^{2}T_{0}\right)\dot{T}-kT^{\prime \prime }\right]}^{\;\cdot \cdot } & ={\left[\varrho c\dot{T}-kT^{\prime \prime }\right]}^{\prime \prime } \end{array}$$

[no detuning of ϱc on the r.h.s.]. It is revealed only in the full 3D treatment, which reveals possible pitfalls of 1D considerations in general as well.

As conclusion of this section, thermal expansion coupled elasticity may introduce a few percent effect (a material dependent but sample size independent value) in determining thermal diffusivity from flash experiments or other transient processes (while its other consequences may be negligible).

## 4. Pseudo-Temperature Approach

The experimental results serve to check whether a certain theory used for describing the observed phenomenon is acceptable or not. The heat pulse (flash) experiment results may show various temperature histories. Generally, the flash measurement results are according to the Fourier theory. In some cases, as reported in [39,40] the temperature histories show “irregular” characteristics, especially these histories could be described by the help of various non-Fourier models [7,34,45,46]. Some kind of non-Fourier behavior could be constructed as it is shown in the following. This is only an illustration how two parallel Fourier mechanisms could result a non-Fourier-like temperature history. The idea is strongly motivated by the hierarchy of Fourier equations in the GK model [44] as mentioned previously; however, their interaction is not described in detail.

The sample that we investigate now is only a hypothetic one, we may call it as a “pseudo-matter”. We consider in the following that the pseudo-matter formed by parallel material strips is wide enough that the interface effects might be neglected, i.e., they are like insulated parallel channels. We also consider that only the thermal conductivities are different, and the strips have the same mass density and specific heat. During the flash experiment after the front side energy input, a simple temperature equalization process happens in the sample in case of adiabatic boundary conditions. Since the flash method is widely developed, the effects of the real measurement conditions (heat losses, heat gain, finite pulse time, etc.) are well treated in the literature.

**Figure 6:**
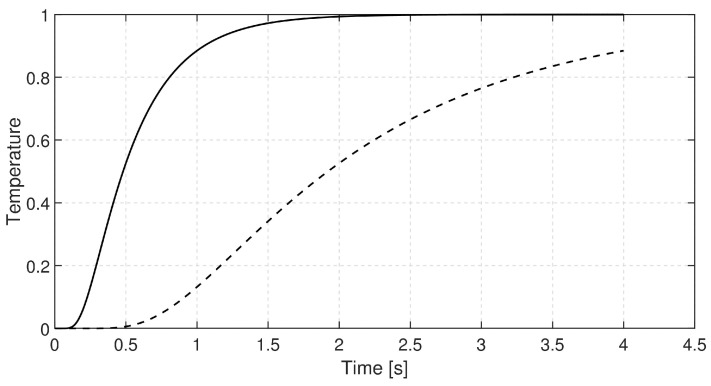
Rear-side temperature history; solid line: $a=10^{-6}\;m{}^{2}/s$, dashed line: $a=2.5\cdot 10^{-7}\;m{}^{2}/s$, L=2mm.

Figure 6 shows two temperature histories with thermal diffusivities of different magnitude, both are the solution of Fourier heat equation.

The mathematical formula that expresses the temperature history of the rear side in the adiabatic case is [47]:

(31) $$\begin{array}{c} \nu \left(\xi =1,Fo\right)=1+2\sum_{m=1}^{\infty }{\left(-1\right)}^{m}e^{-(m^{2}\pi^{2}Fo)}, \end{array}$$

where ν is the dimensionless temperature, i.e., $\nu =\frac{T-T_{0}}{T_{\max}-T_{0}}$, where $T_{0}$ is the initial temperature and $T_{\max}$ is the asymptotic temperature corresponding to equilibrium with adiabatic boundary conditions, ξ is the normalized spatial coordinate (ξ=1 corresponds to the rear-side) and $Fo=a\cdot t/(L^{2})$ stands for the Fourier number (dimensionless time variable). This is an infinite series with property of slow convergence for short initial time intervals. An alternative formula derived using the Laplace theorem to obtain faster convergence for Fo<1 [48]:

(32) $$\begin{array}{c} p\left(Fo\right)=\frac{2}{\sqrt{\pi Fo}}\sum_{n=0}^{\infty }e^{-\frac{{\left(2n+1\right)}^{2}}{4Fo}}, \end{array}$$

wherein *p* is the Laplace transform of ν. In the further analysis we use Equation (32) to calculate the rear-side temperature history.

**Figure 7:**
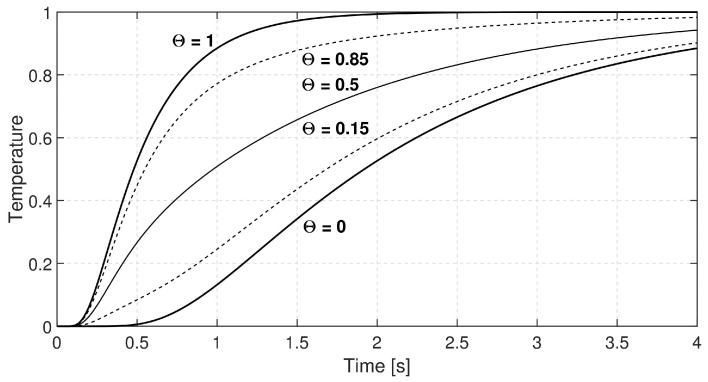
Rear-side temperature histories.

So far, we described two parallel heat-conducting layers without direct interaction among them; however, let us suppose that they can change energy only at their rear side through a very thin layer with excellent conduction properties. Eventually, that models the role of the silver layer used in our experiments to close the thermocouple circuit and assure that we measure the temperature of that layer instead of any internal one from the material. Actually, the silver layer averages the rear-side temperature histories of the parallel strips. We considered the mixing of temperature histories using the formula:

(33) $$\begin{array}{c} p\left(Fo\right)=\Theta p_{1}\left(a=10^{-6}\;m{}^{2}/s,Fo_{1}\right)+\left(1-\Theta \right)p_{2}\left(a=2.5\cdot 10^{-7}\;m{}^{2}/s,Fo_{2}\right), \end{array}$$

that is, taking the convex combination of different solutions of Fourier heat Equation (5). Figure 7 shows a few possible cases of mixing.

## 5. Outlook and Summary

This pseudo-material virtual experiment is only to demonstrate that there might be several effects causing non-Fourier behavior of the registered temperature data. Here, the assumed mixing of “Fourier-temperatures” is analogous with the GK equation in sense of the hierarchy of Fourier equation: dual heat-conducting channels are present and interact with each other. However, the GK equation is more general, there is no need to assume some mechanism to derive the constitutive equation.

Comparing Equations (6) to (25), the hierarchy of Fourier equation appears in a different way. While (6) contains the zeroth and first order time derivatives of Fourier equation, the (25) instead contains its second order time and spaces derivatives. Recalling that Equation (25)

(34) $$\begin{array}{cc} \frac{1}{c_{\|}^{2}}{\left(\gamma_{1}^{}\dot{T}-k▵T\right)}^{\;\cdot \cdot } & =▵[\underset{\gamma_{2}^{}}{\left(\underset{︸}{\varrho c-\frac{6E^{d}E^{s}\alpha^{2}T_{0}}{E^{s}+2E^{d}}}\right)}\dot{T}-k▵T]\;. \end{array}$$

is derived using the assumption that thermal expansion is present beside heat conduction, it becomes obvious to compare it to a ballistic (i.e., thermal expansion induced) heat conduction model. Let us consider such model from [7]:

(35) $$\begin{array}{c} \tau_{1}\tau_{2}\dddot{T}+\left(\tau_{1}+\tau_{2}\right)\ddot{T}+\dot{T}=a▵T+\left(\kappa^{2}+a\tau_{2}\right)▵\dot{T}, \end{array}$$

where $\tau_{1}$ and $\tau_{2}$ are relaxation times. Equation (35) have been tested on experiments, too [16]. Eventually, the GK equation is extended with a third order time derivative and the coefficients are modified by presence of $\tau_{2}$. On contrary to Equation (34), it does not contain any fourth order derivative. Actually, the existing hierarchy of Fourier equation is extended, instead of τ and $\kappa^{2}$ the terms $\left(\tau_{1}+\tau_{2}\right)$ and $\left(\kappa^{2}+a\tau_{2}\right)$ appear within (35).

Although it is still not clear exactly what leads to over-diffusive heat conduction, the presented possible interpretations and approaches can be helpful to understand the underlying mechanism. It is not the first time to experimentally measure the over-diffusive propagation, but it is to consider its size dependence. The simplest thermo-mechanical coupling predicts size dependence of material coefficients that can be relevant in certain cases. All three approaches lead to a system of partial differential equations, which can be called hierarchical.

## Figures and Tables

**Table 1:** Summarized results of fitted coefficients in Fourier and GK equations.

Thickness *L*, [mm]	Fourier Thermal Diffusivity $a_{F}$,$\cdot 10^{-6}\left[\frac{m^{2}}{s}\right]$	Guyer-Krumhansl Thermal Diffusivity $a_{\mathrm{GK}}$, $\cdot 10^{-6}\left[\frac{m^{2}}{s}\right]$	Relaxation Time τ, [s]	Dissipation Parameter $\kappa^{2}$, $\cdot 10^{-6}\left[m^{2}\right]$
1.86	0.62	0.55	0.738	0.509
2.75	0.67	0.604	0.955	0.67
3.84	0.685	0.68	0.664	0.48

**Table 2:** Ratio of the fitted coefficients.

Thickness *L*, [mm]	Ratio of Parameters $\frac{a_{\mathrm{GK}}\tau }{\kappa^{2}}$
1.86	0.804
2.75	0.854
3.84	0.943
